# A versatile large-scale coherent Ising machine from microwave to visible and telecom wavelength bands

**DOI:** 10.1038/s41377-026-02225-5

**Published:** 2026-02-24

**Authors:** Haoran Zhang, Jian Li, Haotao Zhu

**Affiliations:** School of Electrical and Electronic Engineering, Nanyang Technological University, Singapore, Singapore

**Keywords:** Quantum optics, Nonlinear optics

## Abstract

A versatile coherent Ising machine platform based on degenerate optical parametric oscillators has emerged as a powerful approach for solving NP-hard combinatorial optimization problems across diverse applications. Recent work demonstrated breakthroughs using femtosecond laser pumping, achieving a 55% success rate for 100-vertex Max-Cut problems with stable performance maintained for over 8 h. This versatile platform supports fully connected problem mapping and has been successfully validated across multiple domains including molecular docking and credit scoring, substantiating the practical potential of optical approaches for large-scale optimization and paving the way for broad integration into industrial applications.

## Abstract (web summary)

A coherent Ising machine platform demonstrates versatile applicability across NP-hard combinatorial optimization problems, supporting the feasibility of optical approaches to large-scale optimization.

## Graphical abstract

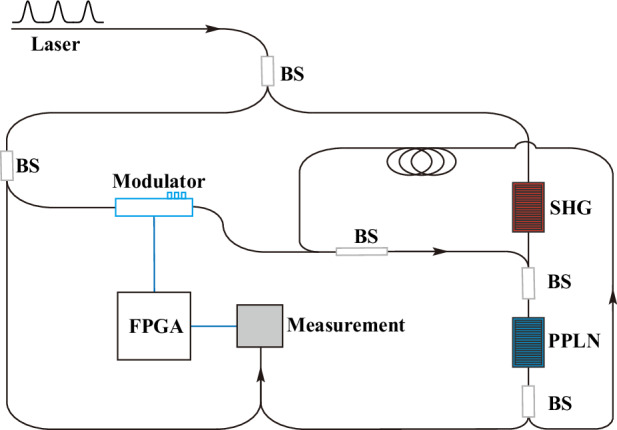

The Ising model plays a crucial role in statistical physics and computational science^1^. Originally developed to describe magnetic ordering, it has evolved into a paradigmatic framework for the study of spin glasses, which are disordered systems characterized by competing interactions and highly rugged energy landscapes. Beyond condensed matter physics, the problem of finding the ground state of an Ising Hamiltonian has wide-ranging applications in computer science, biology, and information processing. Many such problems can be formulated by quadratic unconstrained binary optimization (QUBO), as numerous combinatorial optimization problems can be naturally reformulated as QUBO and then mapped onto equivalent Ising models. From the perspective of computational complexity, the Ising problem belongs to the NP-hard class, posing significant challenges for classical algorithms at large scales.

To address such problems, a variety of heuristic and physics-inspired approaches have been explored. Simulated annealing exploits thermal fluctuations to escape local minima, while quantum annealing and quantum adiabatic computation aim to leverage quantum tunneling effects to improve optimization performance. Experimental implementations of quantum annealing have been demonstrated in several physical platforms, including superconducting circuits, magnetic systems, and nuclear magnetic resonance setups. While these approaches have achieved notable progress, practical implementations often face constraints in physical connectivity, which can introduce additional overhead when encoding large or densely connected problems^2^.

Motivated by these challenges, alternative physical realizations of Ising machines have been proposed. Among them, optical systems provide a particularly attractive platform. Lasers and degenerate optical parametric oscillator (DOPO) are open, dissipative systems that undergo second-order phase transitions at their oscillation thresholds, naturally supporting collective dynamics and competition among modes. In laser networks, gain competition and cross-saturation favor oscillation modes with minimal threshold gain, a mechanism that can be engineered to reflect the structure of an Ising Hamiltonian. As a result, the system tends to evolve toward low-energy spin configurations^3^.

DOPOs offer an especially natural representation of Ising spins, as each oscillator above threshold adopts one of two stable phase states^4^. Building on this property, coherent Ising machines (CIMs) based on time-multiplexed DOPO have been experimentally demonstrated^5,6^ (Fig. 1). By combining optical coherence with measurement-and-feedback schemes^7^, CIMs enable flexible and scalable implementation of spin–spin interactions^8^, including all-to-all connectivity^9^. This capability allows arbitrary graph-based optimization problems to be mapped directly onto the hardware without the need for complex embedding procedures^10^. Owing to their intrinsic parallelism, fast optical dynamics, and high connectivity, CIMs represent a promising and versatile platform for large-scale combinatorial optimization.

**Fig. 1:**
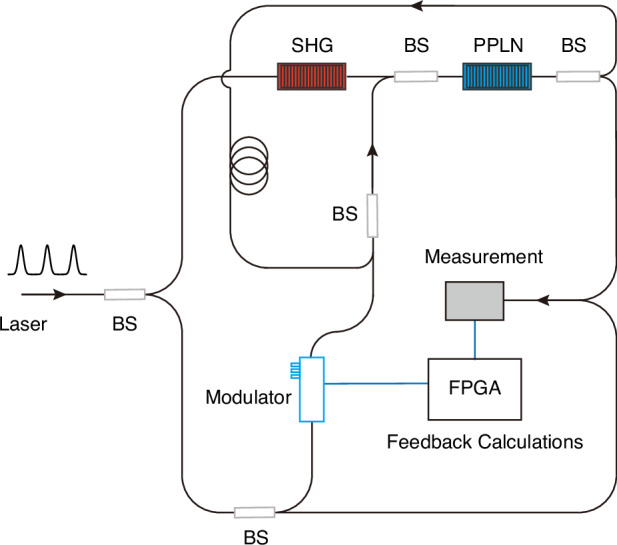
Schematic of a Coherent Ising Machine (CIM) based on a degenerate optical parametric oscillator (DOPO) with measurement and feedback. A mode-locked pulsed laser provides ultrashort pump pulses that are split and injected into an optical loop containing a second-harmonic generation (SHG) stage and a periodically poled lithium niobate (PPLN) crystal, enabling DOPO in a ring cavity. Above the oscillation threshold, each circulating pulse acquires one of two stable phase states, which naturally encode binary Ising spins. Time-multiplexing allows a large number of optical pulses to circulate in the same cavity, forming a scalable network of interacting spins. A fraction of the DOPO output is extracted by beam splitters (BS) and measured via balanced homodyne detection, providing access to the phase of each optical pulse. The measurement results are digitized and processed by a field-programmable gate array (FPGA), where the effective spin–spin coupling strengths are calculated according to the target Ising Hamiltonian. The resulting feedback signals are then applied to the optical loop through modulators, completing a measurement-and-feedback coupling scheme. This architecture enables programmable interactions, including all-to-all connectivity, among time-multiplexed optical spins, allowing arbitrary combinatorial optimization problems to be mapped directly onto the CIM hardware

Building on these foundational principles, significant experimental progress has been achieved recently. In 2022, Cen et al. demonstrated a large-scale CIM based on optoelectronic parametric oscillators at microwave wavelengths^11^. More recently, in 2026, Wei et al. reported a versatile CIM platform operating at both visible and telecom wavelength bands, marking a substantial advancement in the field^12^.

This work achieved further advances by pumping the DOPOs with femtosecond laser pulses, leading to notable performance improvements. The femtosecond pulses deliver significantly higher peak power, enhancing nonlinearity and amplifying quantum effects while enabling DOPO generation at lower average power levels. This strengthens the quantum noise mechanisms that help explore solution spaces and escape local minima. A key architectural advantage of Wei’s work is the support for arbitrarily connected problem graphs. Unlike platforms with connectivity constraints requiring complex embedding procedures, the all-to-all coupling achieved through measurement-feedback mechanisms allows arbitrary optimization problems to be mapped directly onto the hardware, maximizing resource utilization.

In benchmark tests on the Max-Cut problem defined on a 100-vertex Möbius Ladder graph, which is a typical NP-hard problem, the system achieved an average success rate of 55% in identifying optimal solutions—the highest reported among CIMs and comparable quantum technologies. Crucially, the system maintained this performance continuously for over 8 h, demonstrating exceptional operational stability essential for practical deployment. Beyond laboratory demonstrations, the research team established a cloud-based platform providing remote access via a software development kit, enabling users across industries to validate solutions for real-world applications. The platform has been successfully applied to molecular docking for drug discovery and credit scoring in financial services, demonstrating its versatility and practical value.

The demonstration of high-accuracy, long-term stable operation, combined with successful real-world applications, substantiates the theoretical promise of CIMs. Current development efforts in fiber optics and integrated photonics point toward clear pathways for scaling these systems to thousands or millions of optical spins on photonic chips. Future research will focus on further stability improvements, hybrid architectures, and algorithms tailored to exploit the unique dynamical properties of optical parametric oscillator networks. As fabrication techniques advance, CIMs are poised to become a valuable complementary technology for tackling large-scale combinatorial optimization challenges across science, engineering, and industry.
